# Multidrug-resistant monophasic *Salmonella* Typhimurium ST34 strain producing *bla*_NDM-5_ carbapenemase isolated from a patient with rectal cancer in Hainan, China: comprehensive whole genome dissection of antibiotic resistance and virulence determinants

**DOI:** 10.3389/fcimb.2026.1942157

**Published:** 2026-10-16

**Authors:** Shan Zhong, Lei Xing, Licheng Wang, Chongzhen Wang, Xiaoying Fu, Lixia Xu, Yuanli Li, Huaxiong Zhu, Lvfen He, Xiong Zhu, Huan Li

**Affiliations:** 1 Clinical & Central Laboratory of Sanya People’s Hospital, Sanya, Hainan, China; 2 Department of Hematology and Oncology of Sanya People’s Hospital, Sanya, Hainan, China

**Keywords:** *bla*_NDM-5_, carbapenemase, monophasic *Salmonella* Typhimurium, multidrug resistance, whole-genome sequencing

## Abstract

**Background:**

Here, we report the identification and comprehensive whole-genome characterization of a New Delhi metallo-β-lactamase 5 (*bla*_NDM-5_)-producing multidrug-resistant (MDR) monophasic *Salmonella* Typhimurium ST34 strain SYA001 from an immunocompromised rectal cancer patient. Monophasic *Salmonella* Typhimurium (*Salmonella* enterica subsp. enterica serovar 1,4,[5],12:i:-) is one of the leading causative agents of foodborne gastroenteritis and hospitalization worldwide, and the rapid emergence and spread of carbapenem-resistant strains have severely compromised clinical treatment and posed a major threat to public health.

**Methods:**

In this study, a series of standardized phenotypic assays were performed to characterize the microbiological, biochemical, and antimicrobial resistance profiles of strain SYA001. Matrix-assisted laser desorption/ionization time-of-flight mass spectrometry (MALDI-TOF MS), biochemical identification, and slide agglutination serotyping were used for species and serotype confirmation. Antimicrobial susceptibility testing was conducted using the VITEK 2 compact system, and six types of carbapenemases were screened via a colloidal gold immunochromatographic assay. Whole-genome sequencing (WGS) was performed on the DNBSEQ-T7 platform, and a comprehensive bioinformatics pipeline was applied to dissect the genomic structure, phylogenetic characteristics, antibiotic resistance determinants, plasmid profiles, prophage regions, *Salmonella* pathogenicity islands (SPIs), and protein-protein interaction (PPI) network of the strain.

**Results:**

Our results confirmed that SYA001 is a serovar 1,4,[5],12:i:- monophasic *Salmonella* Typhimurium belonging to the globally epidemic ST34 clone. The strain exhibited an MDR phenotype, with resistance to 7 classes of antibiotics including carbapenems, cephalosporins, and aminoglycosides, and was only susceptible to aztreonam, amikacin, tigecycline, and colistin. Genomic analysis identified the core carbapenem resistance gene *bla*_NDM-5_, along with multiple coexisting resistance genes including *bla*_OXA-10_, *qnrS*1, *tet*(A), *sul*2, and *sul*3 and so on. Four plasmid replicons (IncHI2, IncHI2A, IncQ1, p0111), 15 prophage regions, and a complete set of virulence-related SPIs (SPI-1 to SPI-5,SPI-9, SPI-13, SPI-14) were detected. PPI network analysis revealed AcrA as the core hub of the efflux-mediated resistance regulatory network.

**Conclusion:**

This study provides genomic insights into *bla*_NDM-5_-producing MDR monophasic *Salmonella.* Typhimurium from an immunocompromised rectal cancer patient, supplements clinical surveillance data of carbapenem-resistant *Salmonella* in Hainan.

## 1 Introduction

*Salmonella*, a genus of Gram-negative bacilli belonging to the family Enterobacteriaceae, is a common zoonotic pathogen that can be transmitted to humans through animals or animal-derived food products (Sánchez-Vargas et al., 2011). Typhoidal *Salmonella* causes enteric fever, while non-typhoidal *Salmonella* (NTS) leads to acute and chronic gastroenteritis. The World Health Organization (WHO) estimates that approximately 550 million people are infected with *Salmonella* annually worldwide. Although the number of cases and deaths from extraintestinal invasive typhoid and paratyphoid infections (14.3 million cases) exceeds those from non-typhoidal and paratyphoid infections (53,500 deaths), the disease burden of infectious diarrhea caused by the latter remains higher (Hald et al., 2016; Kirk et al., 2015; GBD 2017 Typhoid and Paratyphoid Collaborators, 2019). With the global spread of antimicrobial resistance, carbapenem-resistant NTS, especially strains producing New Delhi metallo-β-lactamase (*bla*_NDM_), have emerged as a critical clinical and public health challenge worldwide, as carbapenems are the last-resort antibiotics for severe Gram-negative bacterial infections.

Serotyping of *Salmonella* is primarily based on the composition of somatic (O) and flagellar (H) antigens, and more than 2600 serovars of Salmonella enterica have been documented to date (Hendriksen et al., 2011). *Salmonella* Typhimurium is a common serovar associated with foodborne diseases, with a lethality rate three times higher than that of other serovars (Gao et al., 2023; Sun et al., 2020). Its antigenic formula is (1,4,[5],12:i:1,2), while the monophasic variant has a similar antigenic formula (1,4,[5],12:i:-) but lacks the second-phase flagellar antigen. Due to the high genetic similarity between *Salmonella* Typhimurium and its monophasic variant, the latter is generally believed to have evolved from the *Salmonella* Typhimurium serotype, mostly due to deletion of the *fljB* gene encoding the second-phase flagellin, although some strains fail to express the second-phase flagellum due to sequence insertions, deletions, or genetic recombination in the *fljB* promoter region (Switt et al., 2009). According to the literature on the EnteroBase public database (Zhou et al., 2020), as of September 2019, a total of 6,840 conventional Legacy multilocus sequence typing (Legacy MLST) sequence types of Salmonella had been identified. The China Local Salmonella Genome Database version 2 (CLSGDB v2) (http://nmdc.cn/clsgdbv2) contains 164 *Salmonella* serovars, more than 200 MLST types, and nearly 8,000 genomes, encompassing both typhoidal and non-typhoidal Salmonella (Wang et al., 2022; Liang et al., 2015). In recent years, ST19 and ST34 have been reported as the two predominant epidemic lineages (Ingle et al., 2021; Wu et al., 2025; Kaur et al., 2025). On March 27, 2022, the WHO reported a multinational outbreak of ST34 monophasic *Salmonella* Typhimurium linked to contaminated chocolate products, demonstrating that this pathogen can spread through unusual food vehicles such as chocolate and drawing widespread attention to its prevalence in Chin (Bean and Post, 2014; Larkin et al., 2022; Qin et al., 2022).

The overuse of antimicrobial agents has driven the enhancement of bacterial resistance. In recent years, novel MDR monophasic *Salmonella* Typhimurium strains have emerged rapidly worldwide, causing multiple foodborne outbreaks in Italy, Spain, and the United States, and their detection rate in diarrheal patients in China has been increasing continuously (Barco et al., 2014; Vázquez et al., 2025; Kawakami et al., 2016; Zeng et al., 2021). Therefore, the identification of novel genes conferring antibiotic resistance in these pathogens is crucial for the development of efficient diagnostic and therapeutic strategies. Genomic sequence analysis of MDR, particularly carbapenemase-producing, monophasic *Salmonella* Typhimurium is the key to discovering and tracking novel antibiotic resistance genes (Zankari et al., 2012). In addition, pathogenicity islands (PAIs) and resistance islands (REIs) are important regulatory regions that act synergistically during bacterial infection and directly affect the virulence of *Salmonella* (Hensel, 2004).

To date, genomic data on *bla*_NDM-5_-producing, multidrug-resistant monophasic *Salmonella* Typhimurium isolates from patients with underlying malignant tumors have been limited. This study characterized the genomic features of an *bla*_NDM-5_-producing, multidrug-resistant monophasic *Salmonella* Typhimurium strain, SYA001, isolated from the feces of a rectal cancer patient in Sanya, Hainan. A series of comprehensive analyses were performed, including the identification of plasmids and prophages, the detection of antimicrobial resistance genes (ARGs) essential for understanding antibiotic resistance mechanisms, the screening of *Salmonella* pathogenicity islands (SPIs) and virulence factors central to elucidating virulence mechanisms, and a protein-protein interaction (PPI) analysis to reveal molecular interaction patterns within the pathogen. This study aims to characterize the first *bla*_NDM-5_-positive MDR monophasic *Salmonella*. Typhimurium ST34 isolate recovered from an immunocompromised rectal cancer patient in Hainan, China. We provide comprehensive genomic dissection of resistance determinants, virulence profiles and mobile genetic elements, to supplement the clinical surveillance data of carbapenem-resistant *Salmonella* in this region and to inform the consultation of anti-infective strategies.

## 2 Materials and methods

### 2.1 Fecal culture and bacterial isolation

The strain was isolated from a rectal cancer patient who presented with acute abdominal pain and severe diarrhea without obvious predisposing factors at a hospital in Sanya, Hainan. Fecal samples were collected in sterile containers and immediately transported to the clinical microbiology laboratory, where they were processed in strict accordance with standard clinical microbiology testing protocols. The fecal specimens were inoculated onto Salmonella-Shigella (SS) agar plates (Autobio Diagnostics Co., Ltd., Zhengzhou, China) and incubated aerobically at 35°C with 5% CO_2_ for 24–48 hours. The growth of bacterial colonies on the medium was observed daily. Single colonies with colorless or translucent edges, black centers, and smooth, moist surfaces were picked and purified by repeated streaking on SS agar plates for subsequent bacterial identification and experimental analysis.

### 2.2 Bacterial phenotypic identification

#### 2.2.1 MALDI-TOF MS identification

Purified bacterial colonies were spotted onto a MALDI-TOF MS stainless steel target plate, mixed with 1 μL of α-cyano-4-hydroxycinnamic acid (CHCA) matrix solution (bioMérieux, France), and air-dried at room temperature. The target plate was loaded into the VITEK^®^ MS fully automated mass spectrometry identification system (bioMérieux, France), and data acquisition and bacterial identification were performed according to the manufacturer’s instructions. A confidence score ≥90% was considered a reliable identification result, while a score <90% was considered suspicious and could not confirm the bacterial species (Seng et al., 2009).

#### 2.2.2 Biochemical, serotypic, and carbapenemase identification

The biochemical characteristics of the purified strain were determined using the VITEK 2 compact fully automated microbial analysis system (bioMérieux, France) with the VITEK 2 GN identification card (bioMérieux, France). The bacterial suspension was inoculated onto the identification card, which was then loaded into the instrument. The instrument automatically completed the biochemical reactions, result reading, and calculation of bacterial identification rates. An identification rate ≥95% was considered a high-confidence reliable biochemical identification result (Renaud et al., 2005).

After identification as *Salmonella* by mass spectrometry and biochemical assays, serotyping was performed by slide agglutination using *Salmonella* diagnostic antisera (Ningbo Tianrun Bio-Pharmaceutical Co., Ltd., Ningbo, China) according to the White-Kauffman-Le Minor (Grimont and Weill, 2007) *Salmonella* diagnostic antigen table. A colloidal gold immunochromatographic assay kit (Beijing Jinchuan Biotechnology Co., Ltd., Beijing, China) was used to screen for six types of carbapenemases: *bla*_KPC_, *bla*_NDM_, *bla*_IM_P, *bla*_VIM_, *bla*_OXA-48_, and *bla*_OXA-23_.

#### 2.2.3 Antimicrobial susceptibility testing

Antimicrobial susceptibility testing was performed using the VITEK 2 compact system with the corresponding antimicrobial susceptibility test card, in strict accordance with the Clinical and Laboratory Standards Institute (CLSI) M100-S35 standards (Clinical and Laboratory Standards Institute, 2025) for *Salmonella* and *Shigella* species. The minimum inhibitory concentrations (MICs) of 25 antimicrobial agents belonging to 11 classes against the test strain were determined. The results were interpreted as susceptible (S), intermediate (I), or resistant (R) according to the standard resistance breakpoints recommended by CLSI. Strains resistant to three or more classes of antimicrobial agents were defined as MDR strains.

### 2.3 Whole-genome sequencing, assembly, and annotation

Whole-genome sequencing of the purified strain SYA001 was performed by BIOYI GENE Tech Co., Ltd (Wuhan, China) using the DNBSEQ-T7 high-throughput sequencing platform (MGI, China) to generate 150 bp paired-end reads (Li et al., 2025). First, raw sequencing data were filtered using fastp v0.20.0 software (https://github.com/OpenGene/fastp) to remove low-quality reads (Q < 20), adapters, and contaminating sequences, yielding high-quality clean data. The sequencing quality was then evaluated using FastQC v0.11.8 software (https://github.com/s-andrews/FastQC). Subsequently, *de novo* genome assembly of the clean data was performed using Unicycler v 0.5.1 (parameters: -1 r1–2 r2 --keep 3 --mode normal) software, and the final genome scaffold sequence was obtained after correction and optimization. Python scripts were used to perform genome statistics, including genome size, number of contigs, contig N50, and GC content. For the assembled contigs, coding sequences (CDSs) were predicted using Prodigal (v 2.6.3) software and only complete CDSs were retained. tRNA genes were predicted using tRNAscan-SE (v 2.0), rRNA genes were predicted using RNAmmer (v 1.2), and other non-coding RNAs (ncRNAs) were predicted by searching the Rfam database using Infernal (v 1.1.4), retaining only results with lengths greater than 80% of the corresponding sequences in the database. Finally, the CDSs were extracted and annotated using InterProScan (v 5.73-104.0) to obtain KEGG and COG classification annotations and analyze the biological pathways and functional categories in which the annotated proteins participate (Kanehisa et al., 2014; Galperin et al., 2015). The assembled genome sequence of the SYA001 has been deposited in GenBank under accession number [PRJNA1448033].

### 2.4 Taxonomic identification

Ribosomal multilocus sequence typing (rMLST) was performed on the assembled genome for taxonomic identification. Sequence types (STs) were determined using the PubMLST database (https://pubmlst.org/). The serotype of strain SYA001 was predicted using the sistr_cmd tool (Yoshida et al., 2016). In silico analysis of seven housekeeping genes was conducted via the EnteroBase platform (https://enterobase.warwick.ac.uk/). Whole-genome alignment between SYA001 and the reference strain *Salmonella* enterica serovar Typhimurium LT2 (GCA_000006945.2) was performed using the nucmer/dnadiff pipeline (MUMmer 4.0.0rc1; nucmer --maxmatch, with delta-filter using -1 and -m parameters for one-to-one and many-to-many alignments, respectively). Average nucleotide identity (ANI) and digital DNA-DNA hybridization (dDDH) values were used as independent validation metrics. ANI was calculated using the EZBioCloud platform (https://www.ezbiocloud.net/tools/ani), and dDDH was determined using the online Genome-to-Genome Distance Calculator (GGDC 3.0, https://ggdc.dsmz.de/ggdc.php). The genomes of LT2 and SYA001 were annotated using the Bakta database, and the proteomes of the two strains were clustered using CD-HIT (identity ≥90%) to assess differences in gene content between LT2 and SYA001.

### 2.5 Plasmid-mediated antibiotic resistance gene screening and colocalization analysis

Assembled contigs were analyzed using PlasmidFinder (v 2.1.6) and MobileElementFinder (v 1.1.2) to identify potential plasmids and insertion sequences, with a minimum identity threshold of 95% and a minimum alignment coverage of 60% (Carattoli and Hasman, 2020). Antibiotic resistance genes within the detected plasmid sequences were identified using ResFinder v4.7.2 and CARD v3.2.4 to characterize resistance determinants associated with clinically important antibiotics (Florensa et al., 2022; Alcock et al., 2020). The results were compiled into annotated tables for downstream analyses, and the detected plasmid replicons were colocalized with antibiotic resistance genes to elucidate the role of plasmids in the dissemination of antimicrobial resistance. To determine the co-localization of the *bla*_NDM-5_ resistance gene with the IncHI2 plasmid replicon in SYA001, we performed BLASTn alignment of the assembled contig sequences of SYA001 against the reference IncHI2 plasmid CP162903.1 (pST2742-1, 246,818 bp) carrying *bla*_NDM-5_. This reference plasmid was isolated from a clinical ST34 Salmonella Typhimurium strain 2742 of human fecal origin in Zhuhai, China, in 2023. MLST of the host strain revealed that the allelic profiles of its seven housekeeping genes were identical to those of SYA001, confirming that both strains belong to ST34.

### 2.6 Prophage prediction

The assembled bacterial genome contig file was organized and uploaded to the PHASTEST web server (https://github.com/ansontwk/PhasTest_API) for prophage screening. This analysis identifies prophage element regions and annotates functions of detected prophage genes, including capsid proteins, tail proteins, terminases and integration-related proteins.

Prophage regions are scored as intact, questionable or incomplete by the following criteria:

1. When the count of a specific phage organism accounts for ≥100% of all CDS in the region, the region gets a total score of 150; otherwise methods 2 and 3 are applied.
2. Method 1: When the count of a specific phage organism accounts for >50% of all CDS in the region, the organism is determined as the major potential phage for this region. Calculate percentage of the organism’s protein count multiplied by 100, plus percentage of the organism’s length multiplied by 50, to get the method 2 score.
3. Method 3: Add 10 points for each detected phage-related keyword (capsid, head, integrase, plate, tail, fiber, coat, transposase, portal, terminase, protease, lysin); add 10 points for a region size >30 Kb; add 10 points for ≥40 proteins in the region; add 10 points when phage-related and hypothetical proteins account for >70% of all proteins in the region.

The higher score between method 2 and method 3 is taken as the region’s total score: <70 is marked incomplete, 70–90 is marked questionable, >90 is marked intact.

### 2.7 Prediction of Salmonella pathogenicity islands and virulence factors

SPIFinder v 2.0 software was used to detect *Salmonella* pathogenicity island (SPI) regions in the assembled genome of SYA001, with sequence alignment thresholds set to a minimum of 95% homology and 60% coverage against the *Salmonella* pathogenicity island database (Roer et al., 2016). The output accession numbers were further verified in GenBank, and gene names were included in the analysis.

### 2.8 Protein-protein interaction analysis

The protein–protein interaction network within strain SYA001 was predicted using the STRING database (v 12.5, https://cn.string-db.org/). Based on relevant findings and the antibiotic resistance gene profile of SYA001, a list of proteins encoded by 17 antibiotic resistance-associated genes was compiled (**Supplementary Table S1**) and used as input for protein-protein interaction analysis to resolve functional associations among these proteins, thereby providing a hypothetical framework for the regulatory organization of the resistance system in this strain.

## 3 Results

### 3.1 SYA001 Is an NDM-producing MDR strain

A series of standardized microbiological and biochemical experiments were performed to identify and characterize strain SYA001. On SS agar plates, the strain exhibited typical colony morphology with colorless or translucent edges, black centers, and smooth, moist surfaces. Identification by MALDI-TOF MS and VITEK 2 compact both confirmed that the strain belonged to the genus *Salmonella*. Serotype agglutination testing showed a positive reaction for the antigenic formula (1,4,[5],12:i:-), thus confirming that strain SYA001 is monophasic *Salmonella* Typhimurium. Colloidal gold immunochromatographic assay for carbapenemases demonstrated that strain SYA001 was positive for *bla*_NDM_-type carbapenemase (**Figure 1**). Whole-genome sequencing predicted this enzyme to be a *bla*_NDM-5_ variant.

**Figure 1:**
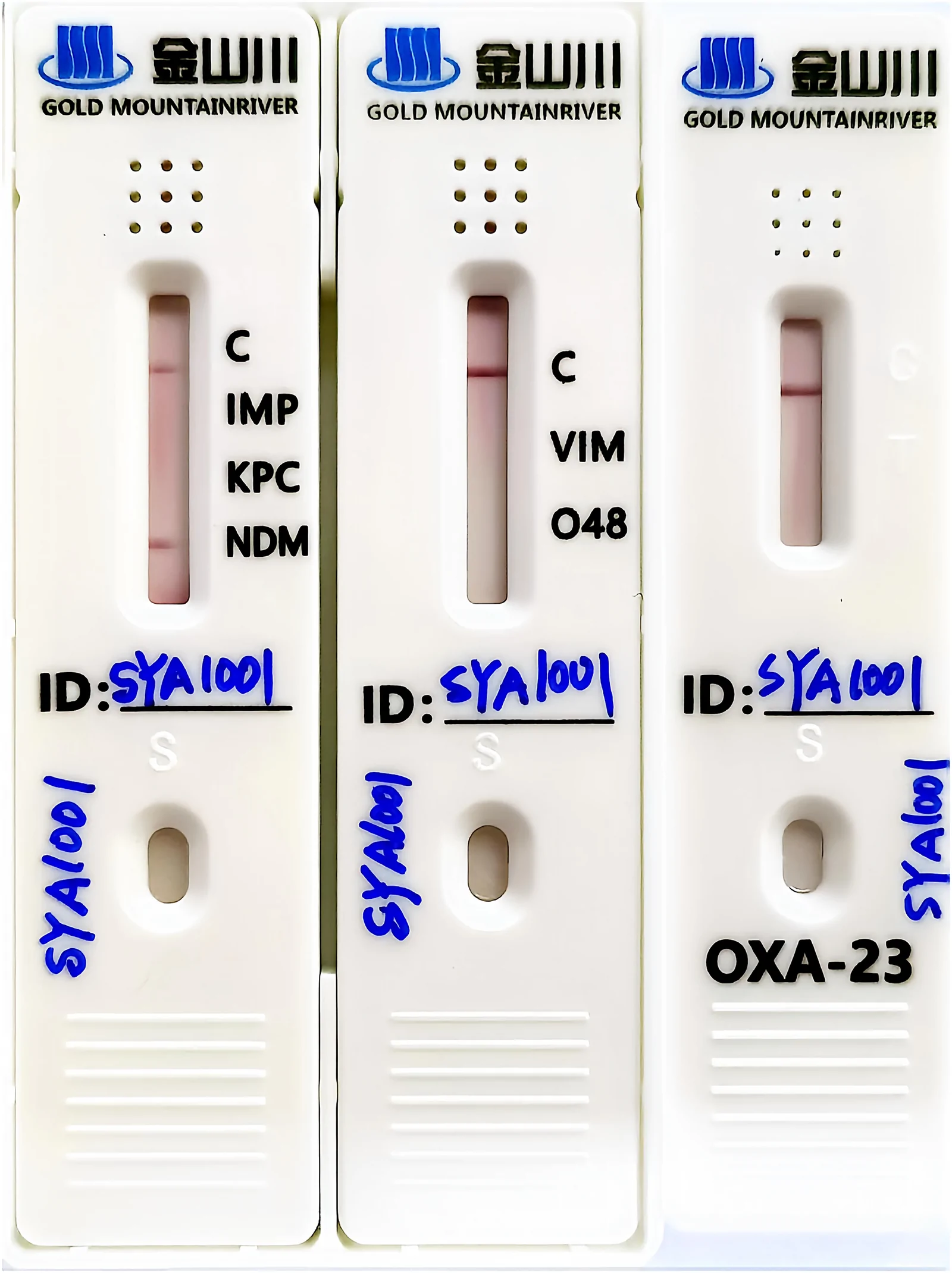
**Figure 1** shows the detection results of six carbapenemases (bla_KPC_, bla_NDM_, bla_IMP_, bla_VIM_, bla_OXA-48_, bla_OXA-23_) in strain SYA001 using the colloidal gold immunochromatography method. The first test strip detects bla_IMP_, bla_KPC_, and bla_NDM_; the second detects bla_VIM_ and bla_OXA-48_; the third detects bla_OXA-23_. A red band in the C line (control line) window of all test strips and one or more red bands in the test area indicate a positive result. The results show that bla_NDM_ on the first test strip of SYA001 is positive, while the other five carbapenemases are negative.

Antimicrobial susceptibility testing was performed for 25 antimicrobial agents across 11 classes. SYA001 exhibited resistance to 7 antibiotic classes including aminopenicillins, β-lactamase inhibitor combinations, cephalosporins, carbapenems, aminoglycosides, tetracyclines, and sulfonamides. It remained susceptible to aztreonam, amikacin, tigecycline, and colistin, and showed intermediate resistance to levofloxacin and ciprofloxacin (**Table 1**). Based on these results, SYA001 was classified as a MDR strain.

**Table 1:** Antibiotic susceptibility of SYA001 using VITEK 2 compact.

Antibiotics	MIC	Interpretation
Aminopenicillins		
Ampicillin	≥64	R
Beta-lactamase inhibitor		
Amoxicillin/Clavulanic acid	≥32	R
Ticarcillin/Clavulanic acid	≥128	R
Piperacillin/Tazobactam	≥128	R
Cefoperazone/Sulbactam	≥64	R
Cephalosporins		
Cefotaxime	≥64	R
Monobactam		
Aztreonam	≤1	S
Cephalosporin 2nd generation		
Cefuroxime	≥64	R
Cefuroxime Axetil	≥64	R
Cephalosporin 3nd generation		
Ceftriaxone	≥64	R
Ceftazidime	≥64	R
Cefotaxime	≥32	R
Cephalosporin 4nd generation		
Cefpixime	≥32	R
Carbapenems		
Imipenem	≥16	R
Meropenem	≥16	R
Ertapenem	≥8	R
Aminoglycosides		
Amikacin	≤2	S
Tobramycin	≥16	R
Quinolone		
Levofloxacin	1	I
Ciprofloxacin	0.5	I
Tetracyclines		
Doxycycline	≥16	R
Minocycline	≥16	R
Tigecycline	1	S
Folate pathway antagonist		
Trimethoprim/Sulfamethoxazole	≥320	R
Polymyxins		
Colistin	≤0.5	S

S, Susceptible; R, Resistance; I, Intermediate.

### 3.2 Genomic and functional analysis of SYA001

The whole-genome sequencing data of SYA001 are summarized in **Table 2**. Raw sequence data statistics showed a data volume of 6.15 G, a total of 46,750,342 reads, a read length of 150 bp, a GC content of 51.01%, a Q20 ratio of 97.14%, and a Q30 ratio of 89.37%. After filtering, 46,747,866 reads were retained for downstream analysis. Genome assembly yielded 89 contigs with a total length of 5,223,782 bp, an N50 value of 222,771 bp, a longest contig of 825,216 bp, a shortest contig of 523 bp, and a GC content of 51.86%. Annotation results revealed 4923 coding sequences (CDSs), 139 miscellaneous RNAs (misc_RNAs), 59 regulatory RNAs, 76 transfer RNAs (tRNAs), and 3 ribosomal RNAs (rRNAs). COG functional classification revealed that 3,903 coding sequences (CDS) were assigned to relevant functional categories, accounting for 79.3% of the total 4,923 annotated protein-coding genes. Among these, carbohydrate transport and metabolism constituted the largest functional category, comprising 446 genes, which represented 9.1% of the total 4,923 annotated protein-coding genes. A total of 384 and 367 genes were classified into the amino acid transport and metabolism and transcription categories, respectively, while 130 genes were annotated to the mobilome (prophages, transposons) category (**Figure 2**). Among the genes annotated in KEGG, a total of 2,613 encoded proteins were classified into six major functional categories, including Cellular Processes, Environmental and Genetic Information Processing, Human Diseases, Metabolism, and Organismal Systems, accounting for 53.1% of the total encoded proteins. Of these, more than half of the annotated encoded proteins (58.9%, 1,538/2,613) were assigned to the Metabolism category. In addition, 235 encoded proteins were annotated to functional categories associated with human diseases. Within this category, the encoded proteins related to bacterial infectious diseases and antimicrobial drug resistance accounted for the highest proportions, at 32.8% and 19.6%, respectively (**Figure 3**).

**Table 2:** The genomic features of monophasic *Salmonella* Typhimurium SYA001.

Type	Number
Raw sequencing data	
Data size (G)	6.15
Read length (bp)	150
Total reads	46,750,342
G+C content (%)	51.01
Q20 (%)	97.14
Q30 (%)	89.37
Clean data (bp)	46,747,866
Assembly sequencing data	
Genome size (bp)	5,223,782
Contig Number	89
Contig N50 (bp)	222,771
Longest contig (bp)	825,216
Shortest contig (bp)	523
G+C content (%)	51.86
Annotation data	
Protein coding genes (CDS)	4923
tRNAs	76
16S rRNA	1
5S rRNAs	1
23S rRNAs	1
Regulatory RNA	59
misc_RNA	139

**Figure 2:**
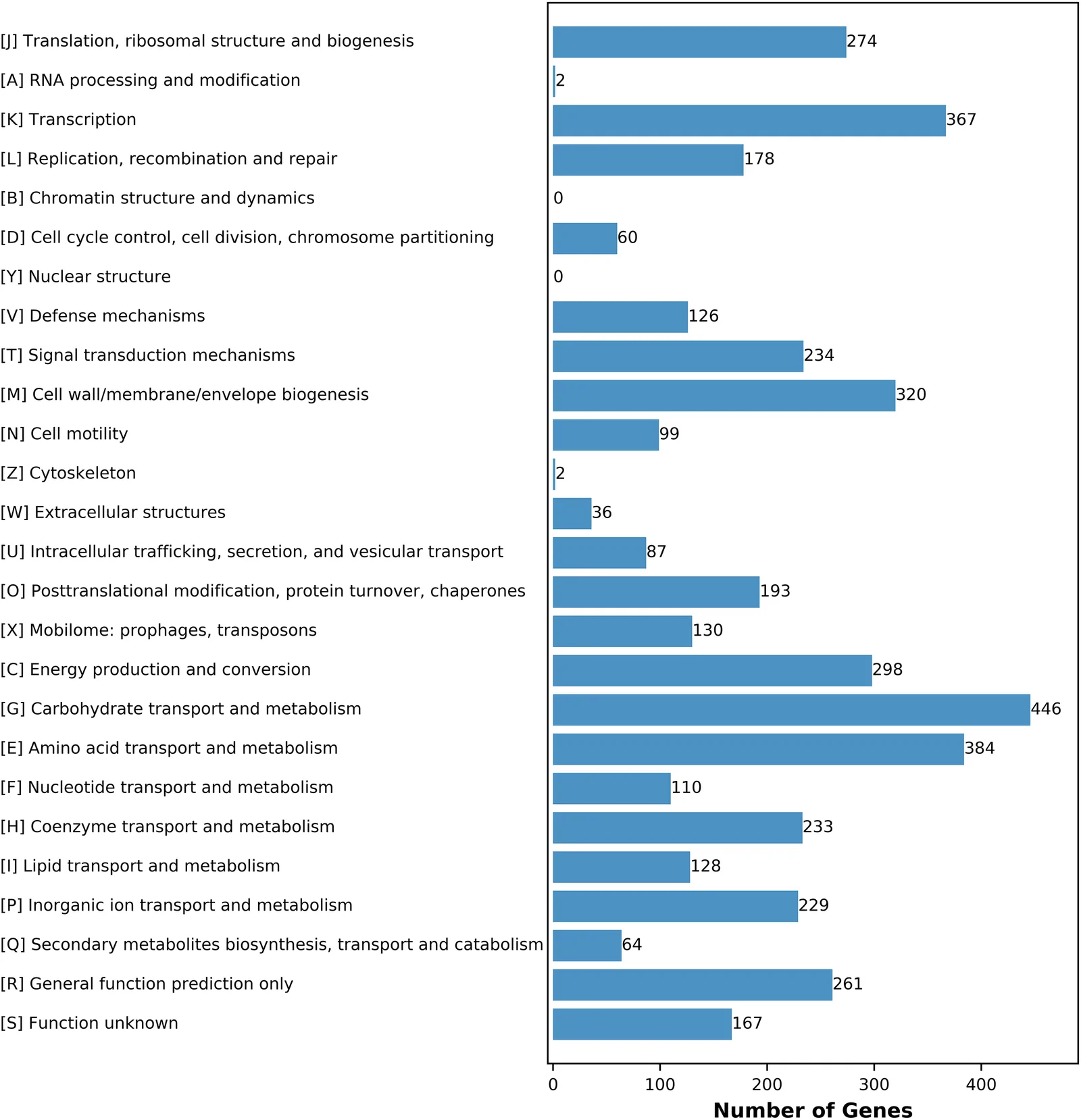
Distribution of COG functional categories of annotated protein-coding genes in SYA001. Genes are grouped into 24 COG categories. The X-axis indicates the number of genes in each category, and the Y-axis indicates COG functional classification.

**Figure 3:**
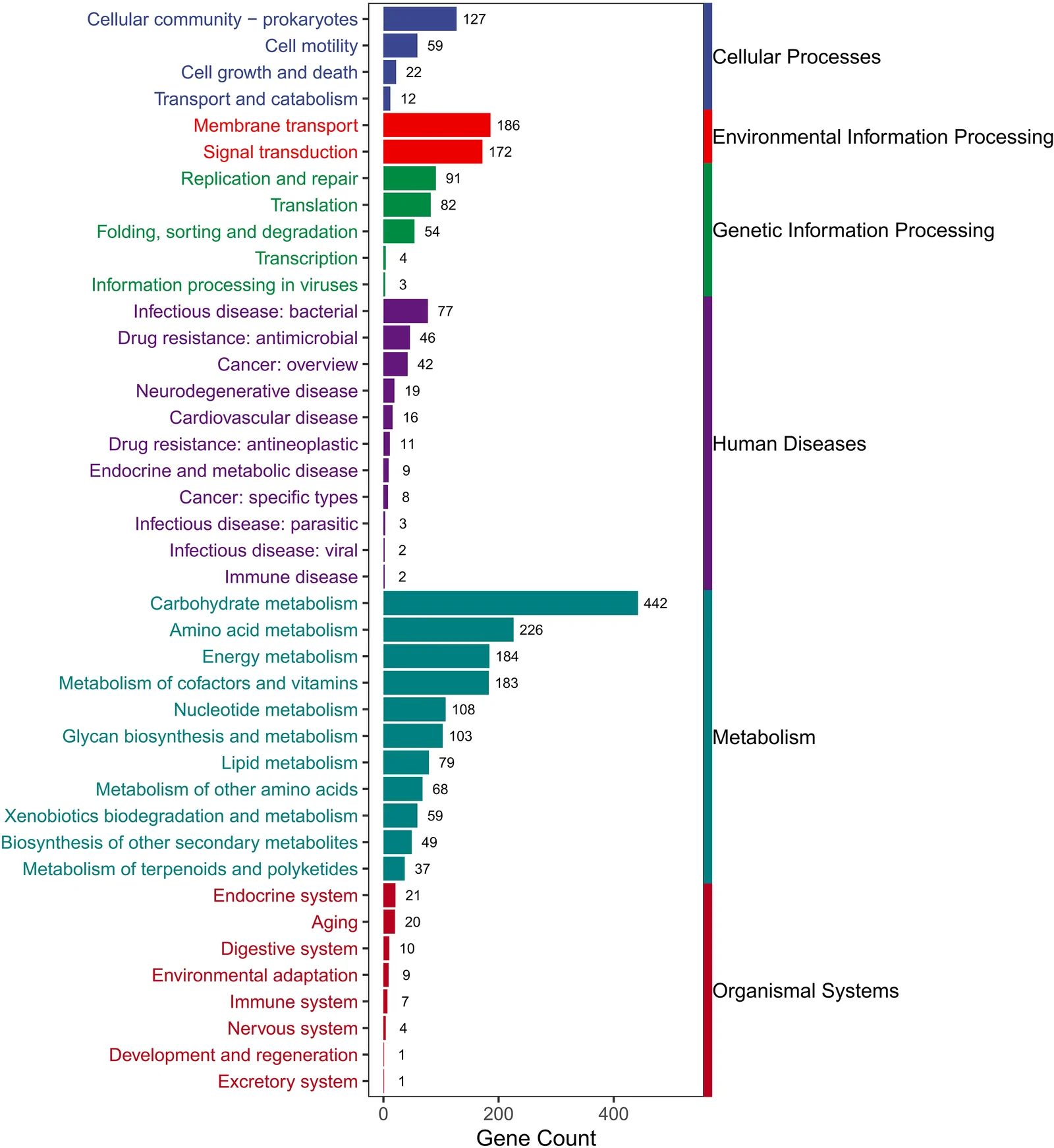
KEGG annotation was mapped to 6 different functional categories, mainly involving cellular processes, environmental information processing, genetic information processing, human diseases, metabolism and organismal systems, among which 235 genes were predicted to be associated with human diseases., the X and Y axes indicate gene count and KEGG pathways, respectively.

### 3.3 In silico Salmonella typing of SYA001

rMLST analysis assigned SYA001’s genome sequence to *Salmonella* enterica. ST analysis based on locus information classified SYA001 as ST34. In silico serotype prediction via the sistr_cmd tool identified the strain as monophasic *Salmonella* Typhimurium (**Supplementary Table S2**). Presence of seven housekeeping genes (*aroC*, *dnaN*, *hemD*, *hisD*, *purE*, *sucA*, *thrA*) in the genome corroborated the MLST typing results (**Supplementary Table S3**). Whole-genome ANI analysis showed the two genomes shared 99.85% homology (**Supplementary Table S4**). dDDH analysis via Formula 2 got a dDDH value of 99.6%. Both values far exceeded the species delineation thresholds of 95% and 70% respectively, confirming the two strains belong to the same species. Further whole-genome comparative analysis showed the average nucleotide identity in aligned regions was 99.96%, with 99.91% overall identity, and only 1,275 single-nucleotide polymorphisms (SNPs) were detected. The two genomes were highly collinear, with no large-scale chromosomal rearrangements. CD-HIT clustering of the two strains’ proteomes obtained 5,240 total gene clusters, 4,301 of which were shared. MLST typing confirmed all seven housekeeping genes were intact in both strains; six alleles were identical, with only dnaN diff*ering (dnaN* - 7 in LT2 v*s dnaN* - 19 in SYA001), consistent with SYA001 belonging to ST34 and LT2 belonging to ST19. The 654 additional gene clusters unique to SYA001 primarily consisted of acquired antimicrobial resistance genes, prophage regions and plasmid-associated mobile genetic elements, with no large deletions of core virulence genes detected.

### 3.4 Prediction and colocalization analysis of antibiotic resistance genes and plasmid-mediated resistance

A total of four plasmid replicons were predicted: IncHI2, IncHI2A, IncQ1, and p011 (**Supplementary Table S5**). Sequence analysis of the corresponding contigs confirmed that the *sul*2 gene is co-localized on the same contig as the IncQ1 replicon, supporting the inference that this plasmid replicon mediates sulfonamide resistance. Genomic analysis identified three categories of antimicrobial resistance-related elements (**Table 3**): (1) 17 acquired antimicrobial resistance genes directly mediating drug resistance, including the core carbapenemase gene *bla*_NDM-5_ and the cephalosporinase gene *bla*_OXA-10_; (2) 18 intrinsic efflux pump structural components encoded by the core chromosome; (3) 7 transcriptional regulatory genes modulating resistance gene expression. Acquired resistance genes were the primary drivers of the multidrug-resistant phenotype of SYA001.

**Table 3:** Classification of antimicrobial resistance-related genomic elements identified in the monophasic *Salmonella* Typhimurium SYA001 genome.

Gene	Identity	Resistance mechanism	AMR gene family	Location
Acquired antimicrobial resistance genes				
*blaNDM-5*	100	Antibiotic inactivation	NDM beta-lactamase	contig000040
*blaMBL*	100	Antibiotic inactivation	Bleomycin resistance protein	contig000040
*blaOXA-10*	100	Antibiotic inactivation	OXA beta-lactamase	contig00072
*AAC(6’)-Iaa*	100	Antibiotic inactivation	Aminoglycoside N-acetyltransferase	contig00001
*APH(3’)-Ia*	98.15	Antibiotic inactivation	Aminoglycoside O-phosphotransferase	contig00042
*APH(4)-Ia*	100	Antibiotic inactivation	Aminoglycoside O-phosphotransferase	contig00042
*AAC(3)-IVa*	99.61	Antibiotic inactivation	Aminoglycoside O-phosphotransferase	contig00042
*APH(6)-Id*	99.64	Antibiotic inactivation	Aminoglycoside O-phosphotransferase	contig00060
*aadA2*	100	Antibiotic inactivation	Aminoglycoside O-phosphotransferase	contig00056
*aadA3*	98.48	Antibiotic inactivation	Aminoglycoside O-phosphotransferase	contig00056
*qnrS1*	100	Antibiotic target protection	Quinolone resistance protein (qnr)	contig00044
*sul2*	99.63	Antibiotic target replacement	Sulfonamide-resistant dihydropteroate synthase	contig00055
*sul3*	100	Antibiotic target replacement	Sulfonamide-resistant dihydropteroate synthase	contig00050
*tet(A)*	99.19	Antibiotic efflux	Major facilitator superfamily (MFS) efflux pump	contig00044
*dfrA14*	99.36	Antibiotic target replacement	Trimethoprim-resistant dihydrofolate reductase	contig00084
*uhpT* (mutant)	95.68	Antibiotic target alteration	Fosfomycin-resistant hexose phosphate transporter	contig00003
*fusA* (EF-Tu mutant)	99.49	Antibiotic target alteration	Elfamycin-resistant elongation factor Tu	contig00026
Intrinsic efflux pump structural components				
*acrA*	91.69	Antibiotic efflux	Resistance-nodulation-cell division (RND) efflux pump	contig00015
*acrB*	94.66	Antibiotic efflux	Resistance-nodulation-cell division (RND) efflux pump	contig00015
*tolC*	94.89	Antibiotic efflux	Outer membrane porin (RND pump adaptor)	contig00001
*acrD*	81.80	Antibiotic efflux	Resistance-nodulation-cell division (RND) efflux pump	contig00023
*acrF*	92.36	Antibiotic efflux	Resistance-nodulation-cell division (RND) efflux pump	contig00001
*emrA*	82.37	Antibiotic efflux	Major facilitator superfamily (MFS) efflux pump	contig00002
*emrB*	95.70	Antibiotic efflux	Major facilitator superfamily (MFS) efflux pump	contig00002
*mdtK*	99.79	Antibiotic efflux	Multidrug and toxic compound extrusion (MATE) transporter	contig00005
*mdsA*	100	Antibiotic efflux	Resistance-nodulation-cell division (RND) efflux pump	contig00009
*mdsB*	100	Antibiotic efflux	Resistance-nodulation-cell division (RND) efflux pump	contig00009
*mdsC*	96.43	Antibiotic efflux	Resistance-nodulation-cell division (RND) efflux pump	contig00009
*golS*	100	Antibiotic efflux	Resistance-nodulation-cell division (RND) efflux pump	contig00009
*kdpE*	91.96	Antibiotic efflux	KdpDE potassium transport system	contig00008
*sdiA*	100	Antibiotic efflux	Resistance-nodulation-cell division (RND) efflux regulator	contig00019
*msbA*	96.39	Antibiotic efflux	ATP-binding cassette (ABC) efflux pump	contig00022
*qacL*	93.64	Antibiotic efflux	Small multidrug resistance (SMR) efflux pump	contig00050
*floR*	99.75	Antibiotic efflux	Major facilitator superfamily (MFS) efflux pump	contig00049
*bacA*	97.07	Antibiotic target alteration	Undecaprenyl pyrophosphate related protein	contig00002
Transcriptional regulatory genes associated with antimicrobial resistance				
*marA*	95.24	Transcriptional regulation	AraC family multidrug resistance activator	contig00001
*crp*	99.05	Transcriptional regulation	cAMP receptor protein (global regulator)	contig00026
*soxS* (mutant)	95.33	Transcriptional regulation	SoxRS oxidative stress regulator	contig00006
*baeR*	94.14	Transcriptional regulation	BaeSR two-component response regulator	contig00005
*baeS*	78.86	Transcriptional regulation	BaeSR two-component sensor kinase	contig00005
*cpxA*	85.08	Transcriptional regulation	CpxAR two-component sensor kinase	contig00017
*emrR*	93.14	Transcriptional regulation	MFS efflux pump repressor	contig00002

Notably, the *bla*_NDM-5_ gene was located on contig000040, while the IncHI2 replicon was predicted on contig000011. Contig assembly of SYA001 revealed that *bla*_NDM-5_ was located on contig000040, whereas the IncHI2 replicon was predicted to reside on contig000011. Annotation of the *bla*_NDM-5_-carrying contig identified a Tn125-type composite transposon resistance gene cassette with the structure IS3000-ISAba125-IS5-*bla*_NDM-5_-*ble*_MBL_ (Hu et al., 2025). Alignment of SYA001 contigs against the reference plasmid CP162903.1 (pST2742-1, 246,818 bp) suggested that *bla*_NDM-5_ was likely located on an IncHI2-type plasmid: 13 SYA001 contigs aligned to this reference plasmid, with a backbone coverage of 96.1% for the union of alignment blocks ≥500 bp with ≥90% identity and 89.7% for the union of alignment blocks ≥2 kb. IS26 was also identified on the third contig (contig000086, 612 bp, 100% identity).

Furthermore, sequence analysis of antibiotic resistance genetic elements in the SYA001 genome revealed that most resistance sequences are associated with efflux pump-mediated antibiotic resistance. A few sequences showed mutations in the elongation factor *EF*-*Tu* and *UhpT* gene, which mutants are conferring resistance to pulvomycin and fosfomycin. It is well established that ST34 clones typically carry a chromosomally integrated ASSuT resistance island encoding classic ampicillin-streptomycin-sulfonamide-tetracycline resistance (Riaz et al., 2026); in strain SYA001, this chromosomal resistance background has been further augmented by acquired plasmid-borne carbapenem resistance determinants.

### 3.5 Prophage sequences in SYA001

The genome of SYA001 (total length: 5,223,782 bp) was predicted to contain 15 prophage regions (**Table 4**). Based on the established scoring criteria for prophage region classification (intact, questionable, or incomplete), the 15 prophage regions were classified as follows (**Figure 4**): 8 intact regions (green regions 2, 6, 7, 9, 10, 11, 13, and 14), 5 incomplete regions (red regions 1, 3, 8, 12, and 15), and 2 questionable regions (yellow regions 4 and 5). A total of 4,986 genes were identified across the whole genome, of which 393 were prophage-associated genes and 4,593 were bacterial genes. The outermost circle depicts the prophage-associated genes annotated according to their functions (**Figure 4**).

**Table 4:** Predicted prophage regions in the SYA001 genome.

Region	Region length (KB)	Completeness (score)	Specific keyword	Region position	Total protein numbers	Phage hit protein numbers	PHAGE SPECIES NUM	Hypothetical protein numbers	Phage+Hypo protein percentage (%
1	9.9	Incomplete (60)	transposase, tail, protease	819114- 829017	11	8	5	2	90.9
2	38.2	Intact (150)	integrase, portal, terminase, capsid, head, tail, plate, protease	965321- 1003597	43	39	14	0	90.6
3	26.8	Incomplete (40)	integrase, protease	2070366- 2097170	24	23	4	0	95.8
4	21.1	Questionable (70)	tail, recombinase, integrase,envelope	2332139- 2353280	11	9	8	0	81.8
5	32.8	Questionable (70)	integrase, protease, plate, transposase	3174553- 3207435	18	11	9	1	66.6
6	55.2	Intact (150)	tail, virion, head, capsid, coat, portal, terminase, lysis,lysin, integrase	3459866- 3515126	60	56	18	2	96.6
7	29.1	Intact (150)	envelope, integrase, capsid, terminase, head, plate, lysin, tail	4044341- 4073538	40	38	9	1	97.5
8	26.6	Incomplete (60)	integrase, lysin, tail, terminase	4227163- 4253781	20	14	11	1	75.0
9	20.4	Intact (120)	lysin, tail, plate	4355611- 4376030	23	22	12	0	95.6
10	27.6	Intact (150)	lysis, terminase, head, portal, capsid, transposase, tail	4989090- 5016779	28	26	6	1	96.4
11	48.6	Intact (150)	tail, head, transposase, plate, terminase	5055080- 5103741	51	49	15	2	100
12	28.6	Incomplete (30)	transposase	5107031- 5135717	23	18	11	0	78.2
13	23.7	Intact (130)	transposase, terminase, head, capsid	5131212- 5154955	35	34	15	0	97.1
14	35.9	Intact (150)	transposase,tail, capsid	5155089- 5191082	41	27	17	2	70.7
15	31.5	Incomplete (60)	integrase,tail	5188487- 5220075	27	19	9	1	74.0

**Figure 4:**
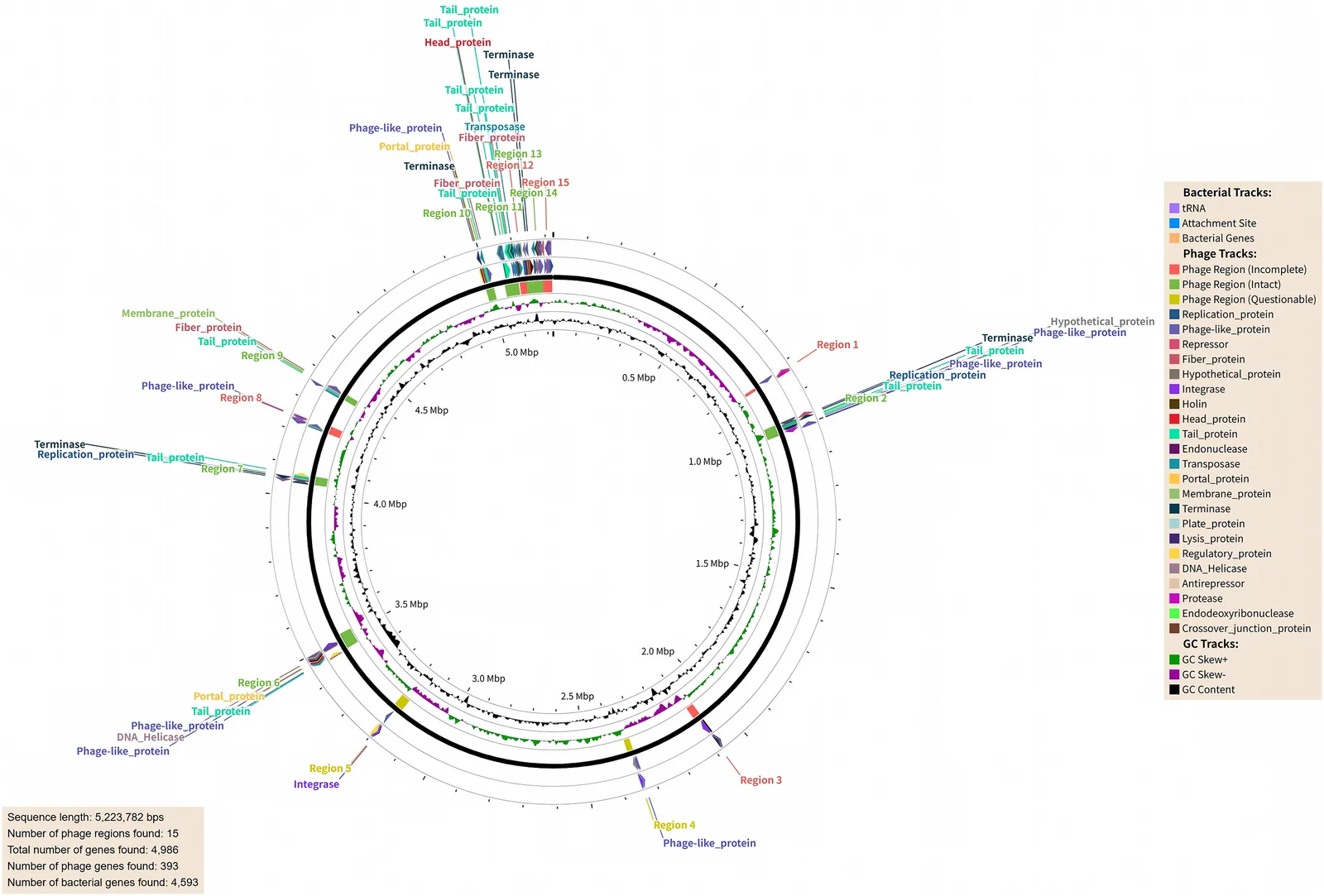
Circular visualization of prophage regions within the SYA001 genome identified by PHASTEST. From the inner to the outer circles are shown: genome size, GC content, GC skew+/GC skew−, and prophage regions; the outermost circle displays the prophage-associated protein gene sets annotated according to predicted functions. A total of 15 prophage-associated regions were predicted in the SYA001 genome (total length: 5,223,782 bp), including 8 intact regions (green regions 2, 6, 7, 9, 10, 11, 13, and 14), 5 incomplete regions (red regions 1, 3, 8, 12, and 15), and 2 questionable regions (yellow regions 4 and 5). Intact prophage regions typically contain structural and regulatory gene sets, including head, tail, virion, capsid, coat, terminase, lysis, lysin, and integrase genes, suggesting potential lytic activity; incomplete prophages lack key structural genes; questionable prophages exhibit limited homology to known phages and require further validation.

### 3.6 *Salmonella* pathogenicity islands in SYA001

Prediction of *Salmonella* pathogenicity islands using SPI Finder 2.0 confirmed the presence virulence-related genomic regions, and the predicted pathogenicity islands are summarized in **Table 5**, including SPI-1, SPI-2, SPI-3, SPI-4, SPI-5, SPI-9, SPI-13 and SPI-14, as well as the C63PI iron uptake island and CS54 island. SPI-1 is a conserved pathogenicity island predicted to mediate bacterial invasion. The SYA001 genome harbors key invasion-associated genes including *invA*, *prgH*, *prgI*, and *prgJ*, which are involved in the assembly of the type III secretion system (T3SS), a key apparatus for injecting virulence factors into host cells. Virulence factors encoded by the regulatory genes *sprB*, *hilD*, and *sipD* play critical roles in coordinating the complex process of pathogen invasion of intestinal epithelium. In addition, the SPI-2 region contains multiple genes involved in the assembly and function of the T3SS, and genes such as *ssrA* and *ssrB* are essential for pathogen survival within host cells.

**Table 5:** The *Salmonella* pathogenicity islands (SPIs) identified in the genome of *Salmonella* Typhimurium SYA001 and their corresponding GenBank references.

SPI	Identity	Contig	Position in contig	Genes	Accession number
SPI-1	100	contig000002	537078-539782	*sprA,sprB*	AF148689
SPI-1	100	contig000002	533989-534428	*prgH,prgI,prgJ*	AY956822
SPI-1	100	contig000002	531889-532318	*hilD*	AY956823
SPI-1	100	contig000002	523141-523610	*sipD*	AY956824
SPI-1	99.04	contig000002	511409-511823	*invA*	AY956825
SPI-1	98.84	contig000002	510271-510529	*invA*	JN982040
SPI-1	99.43	contig000002	497537-500689	*mutS*	U16303
SPI-2	99.22	contig000001	465591-466227	*ssaO,ssaP*	AY956826
SPI-2	100	contig000001	472896-473537	*ssaG,ssaH,ssaI,ssaJ*	AY956827
SPI-2	100	contig000001	468510-468904	*ssaV*	AY956828
SPI-2	99.76	contig000001	475138-475562	*sscB,sseF*	AY956829
SPI-2	99.82	contig000001	485153-485699	*ssrA*	AY956830
SPI-2	100	contig000001	482855-483238	*spiC*	JN673272
SPI-2	100	contig000001	486028-487279	*ssrA,ssrB*	Z95891
SPI-13	99.41	contig000002	271306-271643	*gacD*	AY956832
SPI-13	100	contig000002	271951-272354	*gtrA*	AY956833
SPI-13	100	contig000002	273722-274062	*gtrB*	AY956834
SPI-14	100	contig000013	52587-53087	*gpiA*	AY956835
SPI-14	99.55	contig000013	58163-58603	*gpiB*	AY956836
SPI-3	100	contig000003	46076-46813	*mgtC*	AJ000509
SPI-3	100	contig000003	62835-64348	*selC*	Y13864
SPI-4	100	contig000006	16259-39669	*S*.Typhimurium LT2	NC_003197
SPI-5	100	contig000001	778502-787570	*S*.Typhimurium LT2	NC_003197
SPI-9	98.59	contig000025	29512-42158	*S*.Typhimurium CT18	NC_003198
C63PI	100	contig000002	540358-544357	*sitA,sitB,sitC,sitD*	AF128999
CS54_ island	99.91	contig000014	1-21135	*xseA,shdA,ratC,ratB,ratA,sinI,sinH,yfgK*	AF140550
Not_ named	100	contig000001	480171-480500	*ssaD*	JQ071613

### 3.7 Protein-protein interaction network of antibiotic resistance-related proteins

Protein-protein interaction network analysis, based on previously validated interaction data, predicted a potential functional association network among the resistance determinants, revealing AcrA as a central hub protein (**Figure 5**). This network provides a hypothetical framework for the regulatory organization of the resistance system in this strain. High-confidence interactions were identified between AcrA and AcrB (combined score = 0.999), as well as among TolC, AcrD, and AcrF, collectively forming the core scaffold of multiple efflux systems. Notably, the AcrA-AcrB interaction was robustly supported by multiple lines of evidence, including genomic co-localization, phylogenetic co-occurrence, experimental validation, and literature mining. Beyond the core efflux components, transcriptional regulators such as MarA, BaeR, BaeS, and CRP exhibited multiple interaction sites with efflux genes and auxiliary components.

**Figure 5:**
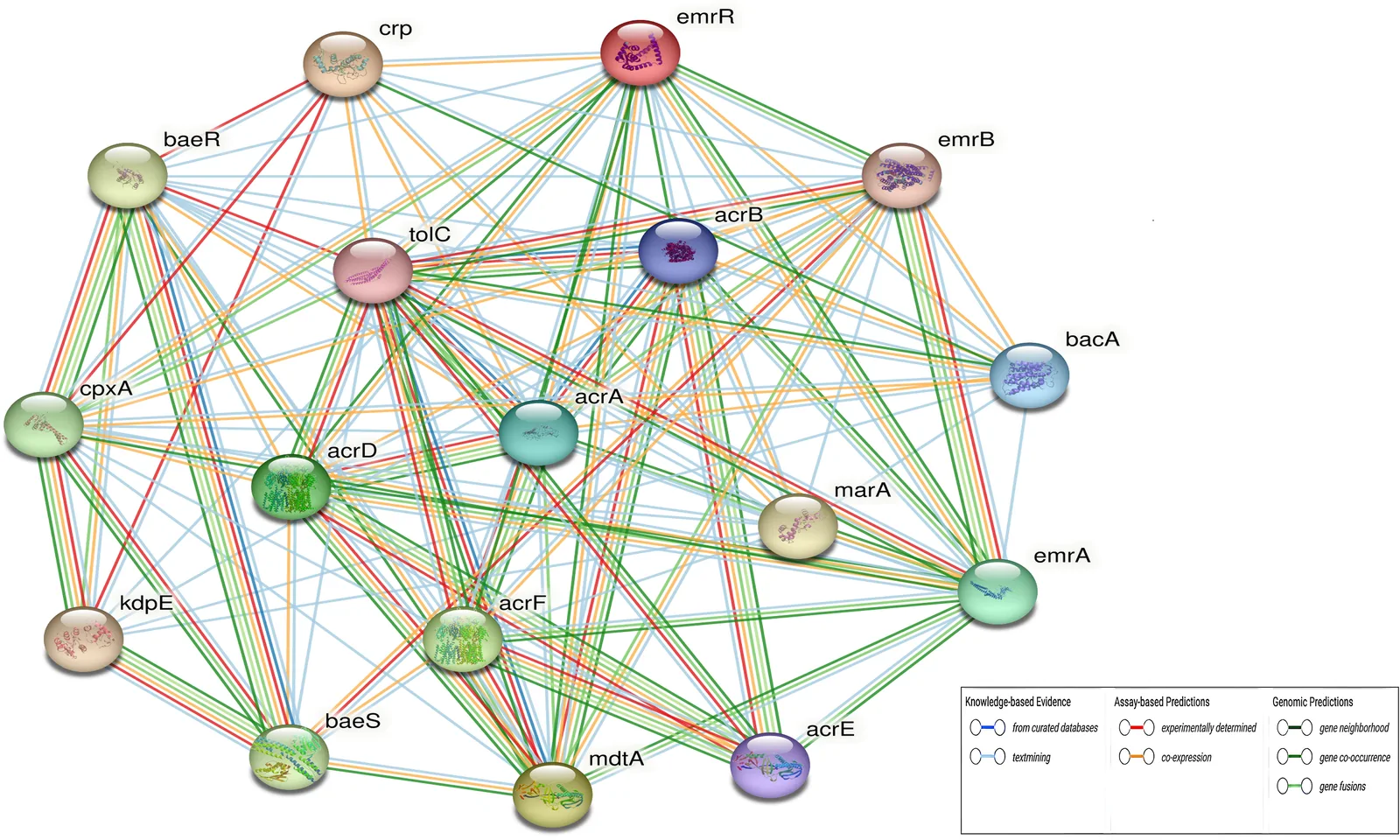
Predicted protein–protein interaction network of antimicrobial resistance-associated proteins constructed using the STRING database (v12.5), with a confidence threshold of ≥0.4. Nodes represent individual proteins. Edges denote functional associations supported by different types of evidence: blue edges represent Knowledge-based Evidence (dark blue: from curated databases; light blue: textmining); red and orange edges represent Assay-based Predictions (red: experimentally determined; orange: co-expression); green edges represent Genomic Predictions (dark green: gene neighborhood; medium green: gene co-occurrence; light green: gene fusions). The core hub protein AcrA is connected to multiple efflux pump components and regulatory proteins, including the classical transporters (AcrB, TolC) and auxiliary transporters (AcrD, AcrF, EmrA, EmrB). Regulatory proteins (MarA, BaeR, BaeS, Crp) also exhibit extensive connectivity with these efflux clusters.

## 4 Discussion

SYA001 was conclusively identified as monophasic *Salmonella* Typhimurium (antigenic formula: 1,4,[5],12:i:-) via MALDI-TOF MS, biochemical testing, and slide agglutination serotyping, consistent with the standard diagnostic criteria for this variant (Switt et al., 2009). rMLST and seven-gene MLST confirmed that SYA001 belongs to ST34, the most prevalent pandemic clone of monophasic *S*. Typhimurium worldwide (Chung The et al., 2023; Wu et al., 2025). Since its first emergence in Spain in the 1990s, the ST34 clone has spread across Europe, America, Asia, and Africa, causing large-scale foodborne outbreaks, underscoring its high transmission capacity and expanding transmission routes (Larkin et al., 2022; Biswas et al., 2019). Our findings add to the growing body of evidence for the continuous acquisition of carbapenem resistance elements in the ST34 pandemic clone, and supplement surveillance data of carbapenem-resistant *Salmonella* in clinical settings.

Whole-genome comparative analysis showed that SYA001 shared more than 99.9% average nucleotide identity with the reference strain *Salmonella* enterica serovar Typhimurium LT2 across aligned regions. Independent verification using ANI and dDDH indicated that their relatedness was far above the 95% and 70% species demarcation thresholds (Kim et al., 2014; Goris et al., 2007), thereby confirming the taxonomic assignment of SYA001. In China, monophasic *Salmonella* Typhimurium has become one of the most common serovars causing diarrheal disease, with detection rates among clinical patients continuing to rise and MDR rates exceeding 70% in most regions (Qin et al., 2022). However, carbapenem-resistant isolates carrying the *bla*_NDM-5_ carbapenemase gene remain relatively rare, making SYA001 an important strain for epidemiological surveillance of antimicrobial-resistant *Salmonella*. The isolation of this strain from a patient with rectal cancer further highlights the need for surveillance in immunocompromised populations, who may serve as high-risk hosts for emerging carbapenem-resistant *Salmonella* clones (Wang et al., 2026).

Antimicrobial susceptibility testing demonstrated that SYA001 exhibited resistance to 7 major classes of antimicrobial agents, including aminopenicillins, β-lactamase inhibitor complexes, cephalosporins, carbapenems, aminoglycosides, tetracyclines, and folate pathway antagonists, while remaining susceptible only to aztreonam, amikacin, tigecycline, and colistin; levofloxacin and ciprofloxacin showed intermediate resistance, fully meeting the definition of MDR bacteria (Clinical and Laboratory Standards Institute, 2025).Whole-genome sequencing identified a comprehensive set of antimicrobial resistance determinants, and the resistance genotypes were generally consistent with the phenotypic resistance profiles. This concordance is in line with findings from previous large-scale validation studies, indicating that whole-genome sequencing-based prediction of antimicrobial resistance genes and phenotypes in SYA001 has high accuracy (Sherry et al., 2023; Kaur et al., 2025).

The core resistance determinant of SYA001 is *bla*_NDM-5_, a highly active variant of the New Delhi metallo-β-lactamase family, which can efficiently hydrolyze almost all β-lactam antibiotics including imipenem, meropenem, and ertapenem, leading to complete resistance to carbapenems-the last-resort drugs for severe Gram-negative bacterial infections (Papp-Wallace et al., 2011; Zhou et al., 2021). However, the isolate remained susceptible to colistin and tigecycline, and this preserved susceptibility provides viable therapeutic options for the clinical management of infections caused by this strain. Nevertheless, continuous surveillance is warranted to track the potential acquisition of mcr genes or other colistin resistance determinants within the ST34 lineage. Colloidal gold immunochromatography confirmed the positive expression of *bla*_NDM_ enzyme, and whole-genome sequencing further verified it as a *bla*_NDM-_5 variant. The *bla*_NDM_ enzymes were first discovered in 2009 and have since spread globally in Enterobacteriaceae, but their prevalence in *Salmonella* is much lower than that in *Klebsiella pneumoniae* and *Escherichia coli* (Bushnell et al., 2013; Walsh et al., 2011; Nahar et al., 2024). Most reported *bla*_NDM_-positive *Salmonella* isolates harbor *bla*_NDM-1_, while strains carrying *bla*_NDM-5_ are rarely identified, particularly among monophasic *Salmonella* Typhimurium (Ke et al., 2024; Hu et al., 2017). Literature has reported that, compared with the more prevalent *bla*_NDM-1_ variant, *bla*_NDM-5_ carries two key amino acid substitutions (Val88Leu and Met154Leu) that confer higher hydrolytic activity against carbapenems and an expanded substrate spectrum. Additionally, *bla*_NDM-5_ exhibits reduced susceptibility to classical β-lactamase inhibitors, which may further compromise treatment options (Yin et al., 2024). While *bla*_NDM-1_ remains the most commonly reported *bla*_NDM_ variant in *Salmonella* globally, *bla*_NDM-5_ has been increasingly identified in clinical isolates in China in recent years, representing an emerging threat to antimicrobial therapy (Deng et al., 2024). In addition to *bla*_NDM-5_, SYA001 carried *bla*_OXA-10_ responsible for cephalosporin resistance, *qnrS*1 mediating quinolone intermediate resistance, *tet(A)* for tetracycline resistance, *sul*2/*sul*3 for sulfonamide resistance, and multiple aminoglycoside-inactivating genes including *AAC*(6’)-*Iaa*, *APH*(3’)-*Ia*, and *AAC*(3)-*IVa*. We also detected mutations in *EF*-*Tu* and *UhpT* genes, which are associated with resistance to elfamycin and fosfomycin, respectively, further enriching the resistance mutation spectrum of *Salmonella* (Zeef et al., 1994; Cattoir et al., 2020).

Antibiotic resistance in Enterobacteriaceae is largely driven by plasmid-mediated horizontal gene transfer (HGT), and plasmids play a decisive role in the rapid evolution and dissemination of MDR clones (Domingues et al., 2025). In this study, four plasmid replicons were identified in SYA001: IncHI2, IncHI2A, IncQ1, and p0111, among which IncHI2 and IncHI2A are highly conserved (identity 100% and 99.52%, respectively). IncHI2 plasmids are recognized as key vectors for the transmission of *bla*_NDM_ genes in Enterobacteriaceae, with broad host ranges, high conjugation efficiency, and the ability to carry multiple ARGs simultaneously, facilitating the cross-species spread of carbapenem resistance genes in clinical and community settings (Algarni et al., 2024). Previous studies have confirmed that IncHI2 plasmids carrying *bla*_NDM-5_ are widely spread in *Salmonella* and *Escherichia coli* in China (Wang et al., 2022; Zeng et al., 2023).

Notably, in the assembled contigs of SYA001, *bla*_NDM-5_ was located on contig000040, whereas the IncHI2 replicon was predicted to reside on contig000011. Comparison with the reference plasmid CP162903.1 suggested that *bla*_NDM-5_ is likely carried on an IncHI2-type plasmid. This discrepancy may be attributable to the inherent limitations of short-read whole-genome sequencing (DNBSEQ-T7, 150 bp paired-end reads): large conjugative plasmids, such as those of the IncHI2 type, contain long repetitive sequences, insertion elements, and high-copy homologous regions that fragment into multiple contigs during *de novo* assembly, making it difficult to reconstruct a single circular plasmid contig from short reads alone (Li et al., 2025; Arredondo-Alonso et al., 2017). Therefore, the findings of this study are consistent with the established epidemiology and molecular characteristics of *bla*_NDM-5_ in China; the distribution of genes across different contigs merely reflects limitations in assembly technology rather than a true absence of *bla*_NDM-5_ from the IncHI2 backbone. IncQ1 is a small broad-host-range plasmid that carries the *sul*2 gene, which directly mediates sulfonamide resistance and enhances the MDR phenotype of SYA001 (Oliva et al., 2017). The coexistence of multiple plasmids in a single strain enables the accumulation and recombination of ARGs, greatly improving the environmental adaptability and drug resistance of bacteria. In SYA001, the *bla*_NDM-5_ gene is located upstream of IS3000-ISAba125-IS5 and immediately downstream of the conserved genetic structure of *ble*_MBL_. This structure represents a Tn125-type composite transposon resistance gene cassette, which facilitates the stable inheritance and expression of resistance genes (Dortet et al., 2012, 2017; Hu X, et al., 2025). Our study revealed the coexistence of IncHI2/IncHI2A, IncQ1, and p0111 plasmid replicons in this *bla*_NDM-5_-positive monophasic *Salmonella*. Typhimurium strain from a rectal cancer patient. The presence of multiple conjugative plasmid replicons suggests a potential for horizontal transmission of carbapenem resistance genes, but this requires further validation by long-read sequencing and conjugation experiments.”

PPI analysis based on the STRING database showed that AcrA is a central hub in the resistance regulatory network of SYA001, forming high-confidence interactions with AcrB, TolC, AcrD, AcrF, EmrA, and EmrB, and collectively constituting multiple resistance-nodulation-cell division (RND) efflux systems, including AcrAB-TolC, AcrEF-TolC, and EmrAB-TolC. In addition to structural efflux genes, global transcriptional regulators such as MarA, BaeR, and CRP also form a hierarchical regulatory network together with efflux pump genes. It has been documented that the AcrAB-TolC system is an important multidrug efflux pump in Gram-negative bacteria and can efflux a variety of antibiotics, including β-lactams, quinolones, tetracyclines, and macrolides, making it a core mechanism of intrinsic and acquired resistance in bacteria (Zhu et al., 2026; Yousefian et al., 2021). It has been reported that MarA can act as a classic activator of the acrAB-tolC operon, not only upregulating efflux pump expression but also downregulating the synthesis of the outer membrane porin OmpF, thereby accelerating drug efflux while reducing antibiotic influx and synergistically enhancing resistance (Middlemiss et al., 2023; Sawant and Shashidhar, 2023). BaeR, as a response regulator of a two-component regulatory system, is activated under environmental stress conditions, thereby inducing the expression of efflux genes such as acrD and further expanding the bacterial resistance spectrum (Baranova and Nikaido, 2002). Genomic interaction analysis suggests a potential synergistic resistance pattern of blaNDM-5 and RND efflux pumps, indicating that the strain may rely on dual strategies of enzyme hydrolysis and active efflux to mediate multidrug resistance. This predicted mechanism requires further experimental validation.

SPIs are core genomic regions that determine the virulence effects of Salmonella, among which SPI-1 and SPI-2 are the core virulence determinants of *Salmonella* (Xiong et al., 2023). The strain SYA001 in this study carries a complete set of pathogenicity islands, including SPI-1, SPI-2, SPI-3, SPI-4, SPI-5, SPI-9, SPI-13, and SPI-14. SPI-1 encodes type III secretion system 1 (T3SS-1), which mediates the invasion of host intestinal epithelial cells by *Salmonella*. The SYA001 genome can be annotated with core invasion genes such as *invA*, *prgH*, *prgI*, *prgJ*, *hilD*, and *sipD*, suggesting that this strain may possess strong adhesion and invasion potential (Lerminiaux et al., 2020; Saleh et al., 2023). SPI-2 encodes type III secretion system 2 (T3SS-2), and the ssrA/ssrB two-component regulatory system annotated in the SYA001 genome belongs to this secretion system; previous studies have confirmed that this system regulates the survival and replication of *Salmonella* within host macrophages, helping the strain evade host immune clearance and mediate invasive infection (Figueira and Holden, 2012; Tomljenovic-Berube et al., 2010). SPI-13 is associated with bacterial metabolic adaptability, and the biological function of SPI-13 in monophasic *Salmonella* Typhimurium remains unclear in current studies; the detection of SPI-13 in SYA001 provides a basis for subsequent analysis of metabolic-related virulence mechanisms of the strain (Elder et al., 2018). In addition, the SYA001 genome also carries the C63PI iron uptake island and the CS54 island, both of which have been reported to participate in regulating bacterial survival *in vivo* and intestinal colonization, enhancing the adaptability of the strain within the host (Kingsley et al., 2003; Zhou et al., 1999). The coexistence of a complete virulence gene profile and multiple drug resistance determinants suggests that this clinical isolate may possess both pathogenic potential and a multidrug-resistant phenotype, posing a potential threat to clinical anti-infective treatment.

Prophages are important mobile genetic elements that drive bacterial genome evolution, virulence acquisition, and the spread of antimicrobial resistance (Chaturongakul and Ounjai, 2014). In this study, a total of 15 prophage regions were identified in the SYA001 genome, including intact, incomplete, and questionable types, encoding 393 prophage-related genes, such as tail proteins, head proteins, portal proteins, integrases, and terminases. Intact prophages possess lysogenic conversion and lytic capabilities and can mediate horizontal transfer of exogenous genes, integrating virulence genes and resistance genes into the bacterial genome, thereby promoting adaptive evolution of bacteria. Previous studies have shown that prophages are widely present in *Salmonella* genomes and are closely associated with the emergence of epidemic clones (Yates et al., 2024). The abundant prophage sequences in SYA001 suggest that this strain may have high genomic plasticity, enabling it to rapidly acquire exogenous genes to adapt to host environments and antibiotic pressure. One of the potential mechanisms underlying the multidrug-resistant phenotype of SYA001 may be that prophages, as potential mobile genetic elements, participate in the acquisition of *bla*_NDM-5_ and other resistance genes and in the regulation of virulence factors. Systematic analysis of the prophage profile of *bla*_NDM-5_-carrying monophasic *Salmonella* Typhimurium provides new insights into the evolutionary mechanisms of this high-risk carbapenem-resistant pathogen.

Rectal cancer patients often have impaired intestinal mucosal barrier and immunosuppression due to surgery, chemotherapy, and tumor progression, making them highly susceptible to foodborne pathogen infections (Li et al., 2023). Infections caused by carbapenem-resistant bacteria in such patients have limited therapeutic options, which are associated with high treatment failure rates and mortality (Katip et al., 2024; Deng et al., 2025). ST34 monophasic *Salmonella* Typhimurium is a typical foodborne pathogen that can be transmitted through food, water, and contact, and has the potential to cause hospital cross-infection (Larkin et al., 2022). Our findings remind clinicians to strengthen the screening of carbapenemase-producing Enterobacteriaceae in immunocompromised cancer patients, especially those with diarrhea symptoms, to achieve early detection, early isolation, and targeted treatment. At the public health level, these preliminary findings suggest the necessity to strengthen surveillance of *bla*_NDM_-producing monophasic *S*. Typhimurium in food, animals and clinical samples in this region. Larger epidemiological studies are required to verify the transmission dynamics of this high-risk clone and guide infection control measures (Li et al., 2025; Laxminarayan et al., 2013).

This study has several limitations. First, this study describes a single clinical isolate from a single center without epidemiological traceability, so the infection source and transmission route of the strain remain unclear, and the broader public health implications require validation in larger sample sizes. Second, short-read whole-genome sequencing cannot resolve complete circular plasmid structures, and thus the definitive genomic location of *bla*_NDM-5_ and its horizontal transmissibility remain to be confirmed by long-read sequencing and plasmid conjugation experiments. Third, *in vitro* cell infection and *in vivo* animal experiments were not conducted to quantitatively evaluate the virulence of SYA001. Fourth, all virulence and resistance mechanism analyses are based on genomic predictions, and actual biological functions require experimental verification. In the future, we will expand the sample size, carry out nationwide traceability surveillance based on genomic epidemiology, perform conjugation experiments, cell models, and animal infection experiments to clarify the transmission dynamics, virulence characteristics, and pathogenic mechanism of this reported strain, and provide more sufficient evidence for the precise prevention and control of carbapenem-resistant monophasic *Salmonella* Typhimurium.

In summary, we report the first genomic characterization of an *bla*_NDM-5_-producing MDR monophasic *Salmonella*. Typhimurium ST34 isolate from a rectal cancer patient in Hainan, China. The strain belongs to the globally epidemic ST34 clone, carries a full set of conserved virulence gene elements, and has acquired multiple antimicrobial resistance determinants including carbapenemase. This study provides important genomic data for the clinical surveillance of carbapenem-resistant *Salmonella*, and highlights the ongoing evolution of antimicrobial resistance in the high-risk ST34 lineage.

## Data Availability

The datasets presented in this study can be found in online repositories. The names of the repository/repositories and accession number(s) can be found in the article/**Supplementary Material**.
