# Supplementary material for: Multidrug-resistant monophasic Salmonella Typhimurium ST34 strain producing blaNDM-5 carbapenemase isolated from a patient with rectal cancer in Hainan, China: comprehensive whole genome dissection of antibiotic resistance and virulence determinants
Source: Front Cell Infect Microbiol. 2026 Oct 16;16:1942157. doi: 10.3389/fcimb.2026.1942157 (PMC13719422; doi:10.3389/fcimb.2026.1942157)
Supplement: Supplementary file 1 [file DataSheet1.docx]

**Supplementary Information (SI)**

**Multidrug-resistant monophasic *Salmonella* Typhimurium ST34 strain producing *blaNDM*-5 carbapenemase isolated from a patient with rectal cancer in Hainan, China: Comprehensive Whole-Genome Dissection of and Antibiotic Resistance Virulence**

**Table S1** Lists the antibiotic resistance genes used as input for the protein-protein interaction analysis

| **Gene** | **Category** | **Function** | **Role in MDR** |
| --- | --- | --- | --- |
| *emrR* | Local regulator | Negative modulator of emrAB operon expression | regulates EmrAB expression |
| *crp* | Global regulator | cAMP receptor protein | Coordinates metabolic homeostasis and mediates indirect regulation of MDR |
| *mdtA* | Efflux pump constituent | Multidrug efflux complex mdtABC. | Participates in antimicrobial efflux |
| *acrF* | Efflux pump constituent | Inner membrane transporter of similar acrB | Multidrug efflux transporter |
| *acrD* | Efflux pump constituent | Multidrug efflux transporter | Mediates aminoglycoside resistance |
| *emrA* | Efflux pump constituent | Membrane fusion protein of EmrAB‑TolC | Promotes export of lipophilic drugs |
| *acrA* | Efflux pump constituent | Periplasmic accessory subunit of AcrAB‑TolC | Component of the major tripartite efflux pump |
| *bacA* | Auxiliary protein | Undecaprenyl-pyrophosphate hydrolase | Resists bacitracin and maintains cell envelope integrity. |
| *acrB* | Efflux pump constituent | Inner membrane component of the AcrAB‑TolC transporter | Multidrug efflux transporter |
| *acrE* | Efflux pump constituent | Periplasmic adaptor protein of the AcrEF-TolC complex | a secondary efflux system like AcrAB‑TolC |
| *tolC* | Efflux pump constituent | Cell envelope outer membrane pore component | Associated with the AcrAB and EmrAB efflux systems |
| *emrB* | Efflux pump constituent | Transporter component of the EmrAB efflux system | Participates in antimicrobial efflux |
| *kdpE* | Two-component regulator | Adaptation‑related regulator | Modulates virulence-associated gene loci |
| *marA* | Global regulator | Transcription-activating factor | Upregulates acrAB operon and stress-related genes |
| *baeR* | Two-component regulator | Transcriptional response regulator | Activates efflux-related genes under stress conditions |
| *baes* | Two-component regulator | Two-component sensor kinase | Constituent of BaeSR-regulated drug efflux |
| *cpxA* | Two-component regulator | Two-component sensor kinase | Triggers CpxR activation and enhances drug efflux |

**Table S2** The serotype parameters predicted for the Monophasic *Salmonella* Typhimurium SYA001 genome sequence using sistr_cmd

| **Type** | **Monophasic *S. Typhimurium* SYA001** |
| --- | --- |
| Antigenic_formula | 1,4,[5],12:i:- |
| H1 antigen prediction(fliC) | i |
| H2 antigen prediction(fljB) | - |
| Mash_serovar | Typhimurium |
| Mash_subspecies | enterica |
| O_antigen | 1,4,[5],12 |
| Predicted_serovar_in_list | Y |
| Serogroup | B |
| Serovar | I 1,4,[5],12:i:- |
| Serovar_antigen | I 1,4,[5],12:i:- |
| Serovar_cgmlst | Typhimurium |

**Table S3** The presence and genomic locations of multi-locus genes in the Monophasic *Salmonella* Typhimurium SYA001 assembly

| **Locus** | **Allele** | **Length** | **Contig** | **Start position** | **End position** |
| --- | --- | --- | --- | --- | --- |
| *aroC* | 10 | 501 | contig000024 | 62176 | 62676 |
| *dnaN* | 19 | 501 | contig000020 | 56421 | 56921 |
| *hemD* | 12 | 432 | contig000016 | 50906 | 51337 |
| *hisD* | 9 | 501 | contig000005 | 81808 | 82308 |
| *purE* | 5 | 399 | contig000015 | 82085 | 82483 |
| *sucA* | 9 | 501 | contig000008 | 184573 | 185073 |
| *thrA* | 2 | 501 | contig000004 | 219450 | 219950 |

**Table S4** Presents the Average Nucleotide Identity (ANI) between Monophasic *Salmonella* Typhimurium SYA001 and the reference strain *Salmonella* Typhimurium LT2

| **Type** | **Monophasic *Salmonella* Typhimurium SYA001** | ***Salmonella* Typhimurium LT2** |
| --- | --- | --- |
| OrthoANIu value (%) | 99.85 | |
| Genome A length (bp) | 5,174,460 |  |
| Genome B length (bp) |  | 4,951,080 |
| Average aligned length (bp) | 3,561,290 | |
| Genome A coverage (%) | 68.82 |  |
| Genome B coverage (%) |  | 71.93 |

**Table S5** The plasmid sequences identified in the Monophasic *Salmonella* Typhimurium SYA001 genome

| **Plasmid** | **Identity (%)** | **Contig** | **Start position** | **End position** | **Accession number** |
| --- | --- | --- | --- | --- | --- |
| IncHI2 | 100 | contig000011 | 101401 | 101727 | BX664015 |
| IncHI2A | 99.52 | contig000011 | 85904 | 86533 | BX664015 |
| IncQ1 | 100 | contig000055 | 1 | 524 | M28829 |
| p0111 | 98.64 | contig000036 | 9724 | 10608 | AP010962 |
